# Distributed Estimation of Fields Using a Sensor Network with Quantized Measurements

**DOI:** 10.3390/s24165299

**Published:** 2024-08-15

**Authors:** Chethaka Jayasekaramudeli, Alex S. Leong, Alexei T. Skvortsov, David J. Nielsen, Omar Ilaya

**Affiliations:** 1 Faculty of Engineering and Information Technology, University of Melbourne, Parkville 3010, Australia; chethakapethmin@gmail.com; 2 Defence Science and Technology Group, Fishermans Bend, Melbourne 3207, Australia; alexei.skvortsov@defence.gov.au (A.T.S.); david.nielsen2@defence.gov.au (D.J.N.); omar.ilaya@defence.gov.au (O.I.)

**Keywords:** distributed estimation, field estimation, quantized measurements, sensor networks, time-varying systems

## Abstract

In this paper, the problem of estimating a scalar field (e.g., the spatial distribution of contaminants in an area) using a sensor network is considered. The sensors are assumed to have quantized measurements. We consider distributed estimation algorithms where each sensor forms its own estimate of the field, with sensors able to share information locally with its neighbours. Two schemes are proposed, called, respectively, measurement diffusion and estimate diffusion. In the measurement diffusion scheme, each sensor broadcasts to its neighbours the latest received measurements of every sensor in the network, while in the estimate diffusion scheme, each sensor will broadcast local estimates and Hessians to its neighbours. Information received from its neighbours will then be iteratively combined at each sensor to form the field estimates. Time-varying scalar fields can also be estimated using both the measurement diffusion and estimate diffusion schemes. Numerical studies illustrate the performance of the proposed algorithms, in particular demonstrating steady state performance close to that of centralized estimation.

## 1. Introduction

In the event of a hazardous incident, e.g., an accidental or malicious release of chemical, biological, radiological, or nuclear (CBRN) materials, the timely acquisition of information related to the incident (position of the source and structure of associated contamination field) is vital in ensuring comprehensive situational awareness, prompt decision-making, and optimal mitigation strategy. In this regard, a well designed sensor system that can rapidly be deployed in the affected area can be considered as a valuable capability in gaining such information.

In recent years, the use of mobile autonomous vehicles (unmanned aerial vehicles (UAVs) or unmanned ground vehicles (UGVs)) with sensors mounted onboard have attracted increasing attention. These vehicles have been employed for hazard source localization/backtracking [1,2,3,4,5,6,7,8,9,10] and inference of field structure [11,12,13,14,15,16,17]. For the case of static or slowly varying scenarios, the application of these vehicles for hazard field estimation and reconstruction has proven to be favourable [11,12,13,14,15,16,17]. The problem becomes more challenging for the case of dynamic estimation when the field changes more rapidly with time. The issue is related to the logistical constraints preventing timely collection of measurements. Indeed, the number of measurements required to estimate the structure of the field in an operational scenario is usually at least on the order of hundreds [11,14,15,17]. Due to the finite speed of the UAVs/UGVs, this may result in a significant time for a vehicle (or vehicles) to travel to different locations and collect the informative measurements. Other potential issues may be related to constraints on the flight time of small UAVs (which is often less than half an hour [13]), or the high operational cost of running multiple UGVs simultaneously [18]. These factors necessitate the investigation of some alternative solutions for the dynamic measurements that can subsequently be used as an input in a data fusion system for inferring the time-varying contaminated field structure. One of the favourable options, considered in the literature, is the application of a variation in sensor networks that can be promptly deployed [19,20] after the hazardous release.

We assume that the sensor network consists of a large number of small, low cost (and possibly disposable) sensor nodes with basic sensing, communication, and computation capabilities (Such sensors are also known as low size, weight, power, and cost (SWaP-C) sensors). In the event of a CBRN incident, many sensors could, for example, be dropped into an affected area from an air platform to form a dynamic sensor network [19,20]. In our study, we consider the scenario where each node calculates its own estimate of the field in a distributed manner, where nodes communicate locally with others within a given communication range. The sensors are assumed to have access only to coarsely quantized measurements, which is motivated by the use of low cost sensors with limited capabilities, as well as the fact that many chemical sensors only give output from a small number of discrete bars [21,22].

Quantization is a nonlinear process which is encountered in many areas such as signal processing [23], wireless communications [24], system identification [25], and feedback control [26]. Quantization is inevitable whenever physical quantities are represented numerically in digital systems [27]. While quantization can offer advantages such as reducing the number of bits required to represent information and hence reduce transmission bandwidth, the nonlinear nature of quantization also makes it challenging in the design of systems and algorithms, especially when the quantization is coarse (i.e., the number of quantization levels is small).

Previous studies on field estimation using sensor networks include [28,29,30,31,32,33,34,35,36]. Distributed estimation schemes are considered in [31,33,35], while [28,29,30,32,34,36] assume centralized estimation using a fusion centre. Poisson distributed measurements are used in [30], binary measurements in [36], general quantized measurements in [34], and noisy (non-quantized) measurements are assumed in [28,29,31,32,33,35]. Furthermore, time-varying fields are considered by assuming a PDE model for the field in [29,36]. However, field estimation algorithms, which are simultaneously (1) distributed, (2) can handle quantized measurements, and (3) can handle time-varying fields, appear to be lacking in the literature, which motivates the current work.

Our approach to field estimation entails approximation of the original field as a weighted sum of radial basis functions [37], where the coefficients of this sum are then estimated. To account for the local communication between neighbouring nodes, two conceptual schemes are considered. In the first scheme, which we call *measurement diffusion*, each node broadcasts to its neighbours the latest received measurement of every node in the network. After receiving the broadcasts from its neighbours, each node will then update their field parameter estimates using the newly acquired measurements via an online optimization approach [38]. In the second scheme, called *estimate diffusion*, each node first forms local (pre-)estimates and Hessians of the field parameters, which are then broadcast to its neighbours. Subsequently, each node will combine the received pre-estimates using an estimate fusion method such as covariance intersection [39] or inverse covariance intersection [40].

The key contributions of this paper are the following:

- We propose novel distributed estimation algorithms that can estimate the global field structure at each node in the sensor network, using quantized measurements and local communications.
- The proposed algorithms are iterative, and can handle time variations in the fields and adjust their estimates accordingly.
- We present comprehensive numerical studies of the algorithms, which demonstrate that the proposed algorithms can achieve steady state performance close to that of centralized estimation.

The paper is organized as follows. Section 2 presents the system model and problem statement. The measurement diffusion scheme is presented in Section 3. A brief overview of estimate fusion is given in Section 4, which serves as a preliminary to the estimate diffusion scheme that is considered in Section 5. Some discussions and extensions of the algorithms can be found in Section 6. Numerical results are given in Section 7. Section 8 draws conclusions.

*Notation*: Given a set X, the cardinality of X is denoted by |X|. A list of some commonly used symbols in this paper is given in Table 1.

## 2. System Model

Consider a region of interest R. We have a field ϕ(.) taking values ϕ(x) at position $x\in R^{2}$. The field ϕ(.) could, for instance, represent the distribution of concentration levels of some contaminant that we are interested in estimating. For notational simplicity, we do not explicitly include time dependence on the field ϕ(.), although our developed algorithms will be able to handle time-varying fields.

We consider a sensor network with *M* nodes located within R. We assume that the nodes are numbered 1,⋯,M and each node knows its own ID. (We note that algorithms exist for assigning each node in a sensor network a unique ID, see, e.g., [41].) The sensor network can be represented as a graph G=(V,E) where V={1,⋯,M} and vertex/node *m* is placed at location $x_{m},\;m=1,\cdots ,M$. We assume that accurate knowledge of the sensor locations can be obtained via GPS. (If GPS is unavailable, then location estimation will also need to be considered [42,43,44].) An edge (m,n) between nodes *m* and *n* exists if $\left|\right|x_{m}-x_{n}\left|\right|<d$ for some communication radius d>0. Given a node *m*, the direct neighbours of *m* are denoted by

$$N_{m}≜\left\{n:|\right|x_{m}-x_{n}||<d,\;n\neq m\}.$$

We will assume that the network is connected.

At time step *k* (in practice, due to response and clear-down times, chemical sensors can obtain new measurements once every few seconds), the sensor at each node *m* will take noisy and quantized measurements $z_{m,k}\left(.\right)$ of the field, with

(1) $$z_{m,k}\left(x_{m}\right)=q\left(\phi \left(x_{m}\right)+v_{m,k}\left(x_{m}\right)\right).$$

Similar measurements models have been considered in, e.g., [21,22].

The term $v_{m,k}\left(.\right)$ in (1) is a noise term, which is assumed to be zero mean. The quantizer q(.) is a quantizer of *L* levels, say {0,1,⋯,L−1}, of the form

(2) $$q\left(x\right)=\left\{\begin{array}{cc} 0, & x<\tau_{0}\\ 1, & \tau_{0}\leq x<\tau_{1}\\ \vdots & \vdots \\ L-2, & \tau_{L-3}\leq x<\tau_{L-2}\\ L-1, & x\geq \tau_{L-2} \end{array}\right.$$

where the quantizer thresholds $\left\{\tau_{0},\cdots ,\tau_{L-2}\right\}$ satisfy $\tau_{0}\leq \tau_{1}\leq \cdots \leq \tau_{L-2}$. The case L=2 corresponds to an ‘on–off’ model with binary measurements, while large *L* can be used to approximate real-valued measurements. Sensor saturation is naturally incorporated into the quantization model. The noise term $v_{m,k}\left(.\right)$ can also be used to take into account false positives (in the case of binary measurements) and uncertain quantizer thresholds [21].

### Problem Statement

We wish to construct an estimate of the field ϕ(.) from the quantized measurements $\left\{z_{m,k}\right\}$ collected by the nodes. Distributed estimation schemes will be considered, where each node will form its own estimate of the field. We assume local communications, so that each node *m* can only transmit/receive information (e.g., measurements or estimates) to/from its direct neighbours $N_{m}$. As we are assuming that measurements will be collected increasingly over time, we also want the estimation algorithms to be iterative, i.e., that the estimates will be progressively updated over time.

In order to estimate the field, in this paper, we consider that the field can be sufficiently well approximated by

(3) $$\phi \left(x\right)\approx \sum_{i=1}^{p}\beta_{i}K_{i}\left(x\right),$$

where *p* is the number of radial basis functions, $K_{i}\left(\cdot \right),i=1,\cdots ,p$ is the *i*-th radial basis function, and $\beta_{i}$ the *i*-th coefficient. Similar models to represent fields have been used in, e.g., [11,12,14,15,16,30,33]. (Other field models considered in the literature include spatial random processes [31,34,45], PDE models [29,32,36], and as a sum of Fourier components [46]). The use of model (3) is motivated by results from approximation theory, which show that many fields can be approximated arbitrarily closely provided a sufficiently large number of basis functions are used [37]. For the basis functions, we will use the Gaussian kernel

$$K_{i}\left(x\right)=\exp\left(-\frac{∥c_{i}{-x∥}^{2}}{\sigma_{i}^{2}}\right),$$

where $c_{i}$ and $\sigma_{i}$ can be regarded as the centre and width, respectively, of the *i*-th basis function.

For a given number of basis functions *p*, we assume that $c_{i}$ and $\sigma_{i}$, i=1,⋯,p, are chosen. (The case where $c_{i}$ and $\sigma_{i}$, i=1,⋯,p, are also estimated can also be considered, but in previous work using mobile sensors, this situation was found to suffer from identifiability issues and sometimes give very unreliable results [15].) Estimation of the field ϕ(.) then becomes a problem of estimating the parameters

$$\beta ≜(\beta_{1},\cdots ,\beta_{p}).$$

In this paper, we will consider two schemes for distributed estimation of the field ϕ(.), which we call measurement diffusion and estimate diffusion, which will be presented in Section 3 and Section 5, respectively. The term ‘diffusion’ is used to convey the idea that information from any node is eventually diffused to the rest of the network via a sequence of local communications between direct neighbours [47,48].

## 3. Measurement Diffusion

In this section, we consider a scheme which we call *measurement diffusion*, where each node will, at each discrete time step *k*, broadcast to its direct neighbours the latest received measurement of *every* node in the network. We assume a single broadcast between two successive time steps, in order to reduce the amount of communication and hence energy usage of the nodes [47,48], noting that there exist other distributed estimation schemes based on consensus algorithms, which can involve multiple rounds of communication between successive time steps [49,50].

**Figure 1:**
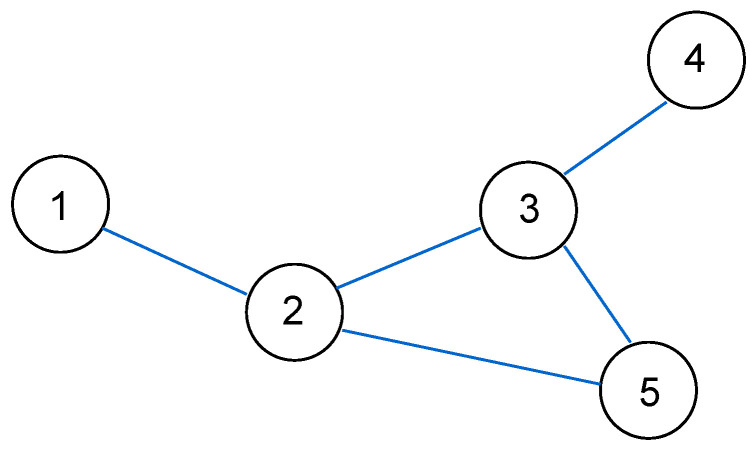
Sensor network example.

Consider a node *m*. At time *k*, measurement $z_{m,k}$ will be broadcast to the nodes in $N_{m}$, since a new measurement has been collected at time *k*. While for a node $n\in N_{m}$, the latest measurement of node *n* available at node *m* at time *k* will be $z_{n,k-1}$, as there is a one hop delay. In general, let L(m,n) denote the length, in terms of the number of hops, of the shortest path between nodes *m* and *n*. Then, under this scheme, assuming that no transmissions are lost, the latest measurement of node *n* available to node *m* at time *k* will be $z_{n,k-L(m,n)}$. As an example, suppose we have the sensor network shown in Figure 1, and consider node 2. Nodes 1, 3, and 5 are one hop away from node 2, while node 4 is two hops away from node 2. Thus, at time *k*, the latest measurements available at node 2 are $z_{1,k-1}$, $z_{2,k}$, $z_{3,k-1}$, $z_{4,k-2}$, and $z_{5,k-1}$. It is not too difficult to see that for a connected network, assuming there are no transmission losses, each measurement from node *m* will eventually reach any other node *n* in the network, with a delay given by the number of hops L(m,n) between nodes *m* and *n*.

Let us be a bit more specific about the communication requirements. At time step *k*, node *m* will broadcast the set

(4) $$C_{m,k}^{\mathrm{latest}}≜\left\{\left(n,k_{n},z_{n,k_{n}}\right):n=1,\cdots ,M\right\}$$

to its direct neighbours, i.e., the node ID, time, and latest measurement of every node is transmitted. Here, $k_{n}$ is the time of the most recent measurement from node *n* available to node *m*, and is equal to k−L(m,n) if no transmissions are lost. The locations $x_{n}$ of the nodes are not transmitted, as we assume that this information has been previously made available to every node beforehand and can be determined given the node ID.

As well as transmitting, each node will also receive the broadcasts of its direct neighbours. At time *k*, after receiving broadcasts of its neighbours, each node *m* will update $C_{m,k}^{\mathrm{latest}}$ to a set $C_{m,k}^{\mathrm{latest}+}$ of latest received measurements of every node, together with their corresponding IDs and times. (At time k+1, the set $C_{m,k}^{\mathrm{latest}+}$ updated with the new measurement $z_{m,k+1}$, will then become $C_{m,k+1}^{\mathrm{latest}}$ and be broadcast by node *m*.) Since the measurements are time-stamped, this update can be performed efficiently. In order to avoid possible double counting, we then further refine $C_{m,k}^{\mathrm{latest}+}$ to a set $C_{m,k}^{\mathrm{new}}$, which contains only new information that have not yet been incorporated into the field estimate of node *m*. This can also be performed efficiently if the times of the last received measurement of each of the nodes are stored [15], and updated whenever a new measurement is received. Note that if there are no transmission losses, then $C_{m,k}^{\mathrm{new}}=C_{m,k}^{\mathrm{latest}+}$.

We next present the estimation procedure for updating the field estimates. The estimation algorithm is based on a computationally efficient online optimization technique [38]. (A sequential Monte Carlo approach can also be used to estimate β [15]; however, the online optimization technique is considerably less computationally intensive, while having similar estimation performance [16].) The aim is to estimate β at node *m* by trying to iteratively minimize a cost function $J_{m,k}\left(.\right)$:

(5) $$J_{m,k}\left(\beta \right)≜\sum_{t=0}^{k}\sum_{\left(n,k_{n},z_{n,k_{n}}\right)\in C_{m,k}^{\mathrm{new}}}g_{n}\left(\beta \right)$$

where the following per stage costs are used:

(6) $$\begin{array}{cc} g_{n}\left(\beta \right)≜\; & \left\{\begin{array}{cc} \log(1+\exp\left(\eta \left(\beta^{T}K\left(x_{n}\right)-\tau_{0}\right)\right)), & z=0\\ \log(1+\exp\left(-\eta \left(\beta^{T}K\left(x_{n}\right)-\tau_{z-1}\right)\right)) & \\ \;+\log(1+\exp\left(\eta \left(\beta^{T}K\left(x_{n}\right)-\tau_{z}\right)\right)), & z\in \{1,\cdots ,L-2\}\\ \log(1+\exp\left(-\eta \left(\beta^{T}K\left(x_{n}\right)-\tau_{L-2}\right)\right)), & z=L-1, \end{array}\right. \end{array}$$

with η>0 being a parameter in the logistic function ℓ(x)≜1/(1+exp(ηx)), and

$$K\left(x\right)≜{\left[\begin{array}{cccc} K_{1}\left(x\right) & K_{2}\left(x\right) & \cdots & K_{p}\left(x\right) \end{array}\right]}^{T}.$$

For notational simplicity. we have also used *z* in place of $z_{n,k_{n}}$. A similar per-stage cost to (6) has been previously shown to be suitable for multi-level quantized measurements [46].

The gradient and Hessian of $g_{n}\left(.\right)$ can be derived as, respectively,

(7) $$\begin{array}{cc} \nabla g_{n}\left(\beta \right)= & \left\{\begin{array}{cc} \frac{\eta }{1+\exp(-\eta \left(\beta^{T}K\left(x_{n}\right)-\tau_{0}\right))}K\left(x_{n}\right), & z=0\\ (\frac{-\eta }{1+\exp(\eta \left(\beta^{T}K\left(x_{n}\right)-\tau_{z-1}\right))} & \\ \;+\frac{\eta }{1+\exp(-\eta \left(\beta^{T}K\left(x_{n}\right)-\tau_{z}\right))})K\left(x_{n}\right), & z\in \{1,\cdots ,L-2\}\\ \frac{-\eta }{1+\exp(\eta \left(\beta^{T}K\left(x_{n}\right)-\tau_{L-2}\right))}K\left(x_{n}\right), & z=L-1, \end{array}\right. \end{array}$$

and

(8) $$\begin{array}{cc} \;\;\;\nabla^{2}g_{n}\left(\beta \right)= & \left\{\;\;\;\begin{array}{cc} \frac{\eta^{2}\exp\left(\eta \left(\beta^{T}K\left(x_{n}\right)-\tau_{0}\right)\right)}{{\left(1+\exp\left(\eta \left(\beta^{T}K\left(x_{n}\right)-\tau_{0}\right)\right)\right)}^{2}}K\left(x_{n}\right)K{\left(x_{n}\right)}^{T}, & \;\;\;z=0\\ (\frac{\eta^{2}\exp\left(\eta \left(\beta^{T}K\left(x_{n}\right)-\tau_{z-1}\right)\right)}{{\left(1+\exp\left(\eta \left(\beta^{T}K\left(x_{n}\right)-\tau_{z-1}\right)\right)\right)}^{2}} & \\ \;+\frac{\eta^{2}\exp\left(\eta \left(\beta^{T}K\left(x_{n}\right)-\tau_{z}\right)\right)}{{\left(1+\exp\left(\eta \left(\beta^{T}K\left(x_{n}\right)-\tau_{z}\right)\right)\right)}^{2}})K\left(x_{n}\right)K{\left(x_{n}\right)}^{T}, & \;\;\;z\in \{1,\cdots ,L-2\}\\ \frac{\eta^{2}\exp\left(\eta \left(\beta^{T}K\left(x_{n}\right)-\tau_{L-2}\right)\right)}{{\left(1+\exp\left(\eta \left(\beta^{T}K\left(x_{n}\right)-\tau_{L-2}\right)\right)\right)}^{2}}K\left(x_{n}\right)K{\left(x_{n}\right)}^{T}, & \;\;\;z=L-1. \end{array}\right. \end{array}$$

We then iteratively estimate the parameters β at node *m* by performing an approximate Newton update similar to [15,46] using the new measurements in $C_{m,k}^{\mathrm{new}}$. The formal description of the measurement diffusion procedure that is run at node *m* is given as Algorithm 1 (each node in the network will perform the same operations), with the equations for the approximate Newton update given in lines 11–14. In Algorithm 1, *G* and *H* represent approximate gradients and Hessians for the cost function (5). The quantity δ in line 12 is a forgetting factor [51] to allow for time-varying fields (where β can vary with *k*) to be estimated. In a slightly different context of sensors mounted on a moving vehicle, previous work in [46] has demonstrated that, for time-varying fields, using a forgetting factor close to but strictly less than 1 allows for quicker tracking of the time-variations than a forgetting factor equal to 1. The quantity ς in line 13 is a Levenberg–Marquardt modification parameter [52] to ensure that the Hessian approximation *H* is invertible at all times.
**Algorithm 1** Field estimation at node *m* using measurement diffusion1:**Algorithm Parameters**: Logistic function parameter η>0, Levenberg–Marquardt parameter ς>0, forgetting factor δ∈(0,1]2:**Outputs**: Parameter estimates $\left\{{\hat{\beta }}_{k}^{\left(m\right)}\right\}$3:Initialize $\tilde{H}=0$, $C_{m,0}^{\mathrm{latest}+}=\emptyset$, and ${\hat{\beta }}_{0}^{\left(m\right)}$4:**for** k=1,2,⋯, **do**5:    Collect a measurement $z_{m,k}$6:    Update $C_{m,k}^{\mathrm{latest}}$ using $C_{m,k-1}^{\mathrm{latest}+}$ and $z_{m,k}$, and broadcast $C_{m,k}^{\mathrm{latest}}$ to neighbours $N_{m}$7:    Receive $\left\{C_{n,k}^{\mathrm{latest}}:n\in N_{m}\right\}$ from neighbours8:    Update $C_{m,k}^{\mathrm{latest}+}$ and construct $C_{m,k}^{\mathrm{new}}$9:    Set $\tilde{\beta }={\hat{\beta }}_{k-1}^{\left(m\right)}$10:    **for** $\left(n,k_{n},z_{n,k_{n}}\right)\in C_{m,k}^{\mathrm{new}}$ **do**11:        $G=\nabla g_{n}\left(\tilde{\beta }\right)$ where $\nabla g_{n}\left(.\right)$ is computed using (7)12:        $\tilde{H}\leftarrow \;\delta \tilde{H}+\nabla^{2}g_{n}\left(\tilde{\beta }\right)$ where $\nabla^{2}g_{n}\left(.\right)$ is computed using (8)13:        $H=\tilde{H}+\varsigma I$14:        $\tilde{\beta }\leftarrow \;\tilde{\beta }-H^{-1}G$15:    **end for**16:    Update ${\hat{\beta }}_{k}^{\left(m\right)}=\tilde{\beta }$17:**end for**

## 4. Estimate Fusion

In this section, we briefly describe the concept of estimate fusion, which will be used later in the estimate diffusion scheme of Section 5. Consider a quantity θ, which we wish to estimate. Suppose two unbiased estimates ${\hat{\theta }}_{A}$ and ${\hat{\theta }}_{B}$ of θ are available, with corresponding estimation error covariances $P_{A}$ and $P_{B}$. We wish to fuse the two estimates ${\hat{\theta }}_{A}$ and ${\hat{\theta }}_{B}$ together to provide an improved estimate. In the case of unknown cross-correlations in the errors, elimination of common information [53] used in obtaining ${\hat{\theta }}_{A}$ and ${\hat{\theta }}_{B}$ is difficult, and one may encounter issues such as double counting, leading to overly confident fused estimates [54]. One way to overcome this issue is to design sub-optimal but consistent fusion rules that over-estimate the true error covariance, for which various different fusion methods have been proposed. In the following, we will describe the covariance intersection [39] and inverse covariance intersection methods [40] methods, which can be used as part of the estimate diffusion scheme to be presented in Section 5, noting that other consistent fusion rules can, in principle, also be considered.

### 4.1. Covariance Intersection

The most commonly used method for computing a consistent estimate when cross-correlations are unknown is covariance intersection [39]. In this method, the fused estimate and associated covariance are computed as

(9) $$\begin{array}{cc} {\hat{\theta }}_{\mathrm{CI}} & =P_{\mathrm{CI}}\left(\omega P_{A}^{-1}{\hat{\theta }}_{A}+\left(1-\omega \right)P_{B}^{-1}{\hat{\theta }}_{B}\right)\\ P_{\mathrm{CI}} & ={\left(\omega P_{A}^{-1}+\left(1-\omega \right)P_{B}^{-1}\right)}^{-1}, \end{array}$$

where the parameter ω∈[0,1] can be chosen to minimize quantities such as the trace or determinant of $P_{\mathrm{CI}}$.

In the case where *N* estimates ${\hat{\theta }}_{1},\cdots ,{\hat{\theta }}_{N}$ with corresponding error covariances $P_{1},\cdots ,P_{N}$ are to be fused, covariance intersection can be extended to (see [55]):

(10) $$\begin{array}{cc} {\hat{\theta }}_{\mathrm{CI}} & =P_{\mathrm{CI}}\left(\omega_{1}P_{1}^{-1}{\hat{\theta }}_{1}+\cdots +\omega_{N}P_{N}^{-1}{\hat{\theta }}_{N}\right)\\ P_{\mathrm{CI}} & ={\left(\omega_{1}P_{1}^{-1}+\cdots +\omega_{N}P_{N}^{-1}\right)}^{-1}, \end{array}$$

where $\omega_{n}\in \left[0,1\right],\forall n$ and $\sum_{n=1}^{N}\omega_{n}=1$.

### 4.2. Inverse Covariance Intersection

Inverse covariance intersection [40] follows a similar principle to covariance intersection, but by considering inverse covariance ellipsoids, it can produce less conservative estimates than covariance intersection. The fused estimate and associated covariance are now computed as

(11) $$\begin{array}{cc} {\hat{\theta }}_{\mathrm{ICI}} & =P_{\mathrm{ICI}}\left(P_{A}^{-1}-\omega {\left(\omega P_{A}+\left(1-\omega \right)P_{B}\right)}^{-1}\right){\hat{\theta }}_{A}\\ & \;+P_{\mathrm{ICI}}\left(P_{B}^{-1}-\left(1-\omega \right){\left(\omega P_{A}+\left(1-\omega \right)P_{B}\right)}^{-1}\right){\hat{\theta }}_{B}\\ P_{\mathrm{ICI}} & ={\left(P_{A}^{-1}+P_{B}^{-1}-{\left(\omega P_{A}+\left(1-\omega \right)P_{B}\right)}^{-1}\right)}^{-1}. \end{array}$$

The parameter ω∈[0,1] can also be optimized to minimize quantities such as the trace or determinant of $P_{\mathrm{ICI}}$. We denote

(12) $$\left({\hat{\theta }}_{\mathrm{ICI}},P_{\mathrm{ICI}}\right)=\mathrm{ICI}\left(\left({\hat{\theta }}_{A},P_{A}\right),\left({\hat{\theta }}_{B},P_{B}\right)\right)$$

as the function that computes and returns $\left({\hat{\theta }}_{\mathrm{ICI}},P_{\mathrm{ICI}}\right)$ using (11).

## 5. Estimate Diffusion

In this section, we will consider an alternative scheme to measurement diffusion, which we call *estimate diffusion*. In this scheme, after a new measurement has been collected at time step *k*, each node *m* will: (1) broadcast its own measurement $z_{m,k}$ and receive measurements $z_{n,k}$ from neighbours $n\in N_{m}$, (2) form a local pre-estimate ${\hat{\beta }}_{k}^{\left(m-\right)}$ (we follow the terminology of [47]) and (approximate) Hessian $H_{k}^{\left(m-\right)}$ using the received measurements, (3) broadcast $\left({\hat{\beta }}_{k}^{\left(m-\right)},H_{k}^{\left(m-\right)}\right)$ and receive $\left({\hat{\beta }}_{k}^{\left(n-\right)},H_{k}^{\left(n-\right)}\right)$ to/from neighbours $n\in N_{m}$, (4) form an updated estimate ${\hat{\beta }}_{k}^{\left(m\right)}$ using the received estimates and Hessians. For example, suppose we again have the sensor network shown in Figure 1, and concentrate on node 2. At time *k*, node 2 will broadcast $z_{2,k}$ to nodes 1, 3, and 5. After receiving $z_{1,k}$, $z_{3,k}$, and $z_{5,k}$ from nodes 1, 3, and 5, respectively, node 2 computes and broadcasts the pre-estimate ${\hat{\beta }}_{k}^{\left(2-\right)}$ and Hessian $H_{k}^{\left(2-\right)}$. After receiving the broadcasts of its neighbours, node 2 will then combine ${\hat{\beta }}_{k}^{\left(1-\right)}$, ${\hat{\beta }}_{k}^{\left(2-\right)}$, ${\hat{\beta }}_{k}^{\left(3-\right)}$, ${\hat{\beta }}_{k}^{\left(5-\right)}$, $H_{k}^{\left(1-\right)}$, $H_{k}^{\left(2-\right)}$, $H_{k}^{\left(3-\right)}$, $H_{k}^{\left(5-\right)}$ in order to form ${\hat{\beta }}_{k}^{\left(2\right)}$. In contrast to measurement diffusion, there are now two transmissions between successive time instances (Steps 1 and 3), which is similar to [47,48]. One could remove the broadcast of measurements in Step 1 and compute ${\hat{\beta }}_{k}^{\left(m-\right)}$ using only $z_{m,k}$; however, we found that this degrades the estimation performance significantly.

The formal description of the procedure that is run at each node *m* is given as Algorithm 2. Similar to Algorithm 1, a forgetting factor δ is used to allow time-varying fields to be estimated. Below, we will provide additional details and explanations of the individual steps.
**Algorithm 2** Field estimation at node *m* using estimate diffusion1:**Algorithm Parameters**: Logistic function parameter η>0, Levenberg–Marquardt parameter ς>0, forgetting factor δ∈(0,1], FusionMethod2:**Outputs**: Parameter estimates $\left\{{\hat{\beta }}_{k}^{\left(m\right)}\right\}$3:Initialize $\tilde{H}=0$ and ${\hat{\beta }}_{0}^{\left(m\right)}$4:**for** k=1,2,⋯, **do**5:    Collect a measurement $z_{m,k}$6:    Broadcast $\left(m,z_{m,k}\right)$ to neighbours $N_{m}$7:    Receive measurements $\left\{\left(n,z_{n,k}\right):n\in N_{m}\right\}$ from neighbours8:    Set $\tilde{\beta }={\hat{\beta }}_{k-1}^{\left(m\right)}$9:    **for** $n\in \left\{m\right\}\cup N_{m}$ **do**10:        $G=\nabla g_{n}\left(\tilde{\beta }\right)$ where $\nabla g_{n}\left(.\right)$ is computed using (7)11:        $\tilde{H}\leftarrow \;\delta \tilde{H}+\nabla^{2}g_{n}\left(\tilde{\beta }\right)$ where $\nabla^{2}g_{n}\left(.\right)$ is computed using (8)12:        $H=\tilde{H}+\varsigma I$13:        $\tilde{\beta }\leftarrow \;\tilde{\beta }-H^{-1}G$14:    **end for**15:    Update pre-estimate ${\hat{\beta }}_{k}^{\left(m-\right)}=\tilde{\beta }$ and $H_{k}^{\left(m-\right)}=H$16:    Broadcast $\left({\hat{\beta }}_{k}^{\left(m-\right)},H_{k}^{\left(m-\right)}\right)$ to neighbours $N_{m}$17:    Receive $\left\{\left({\hat{\beta }}_{k}^{\left(n-\right)},H_{k}^{\left(n-\right)}\right):n\in N_{m}\right\}$ from neighbours18:    **if** FusionMethod is CovarianceIntersection **then**19:        Set $\omega_{n}=1/\left(\right|N_{m}\left|+1\right)$ and compute $P_{\mathrm{CI}}={\left(\sum_{n\in \left\{m\right\}\cup N_{m}}\omega_{n}H_{k}^{\left(n-\right)}\right)}^{-1}$20:        Update ${\hat{\beta }}_{k}^{\left(m\right)}=P_{\mathrm{CI}}\sum_{n\in \left\{m\right\}\cup N_{m}}\omega_{n}H_{k}^{\left(n-\right)}{\hat{\beta }}_{k}^{\left(n-\right)}$ and $\tilde{H}=P_{CI}^{-1}-\varsigma I$21:    **else if** FusionMethod is InverseCovarianceIntersection **then**22:        Set ω=1/2 and $\left({\hat{\beta }}_{A},P_{A}\right)=\left({\hat{\beta }}_{k}^{\left(m-\right)},{\left(H_{k}^{\left(m-\right)}\right)}^{-1}\right)$23:        **for** $n\in N_{m}$ **do**24:           Set $\left({\hat{\beta }}_{B},P_{B}\right)=\left({\hat{\beta }}_{k}^{\left(n-\right)},{\left(H_{k}^{\left(n-\right)}\right)}^{-1}\right)$25:           $\left({\hat{\beta }}_{A},P_{A}\right)\leftarrow \;\mathrm{ICI}\left(\left({\hat{\beta }}_{A},P_{A}\right),\left({\hat{\beta }}_{B},P_{B}\right)\right)$ using (12)26:        **end for**27:        Update ${\hat{\beta }}_{k}^{\left(m\right)}={\hat{\beta }}_{A}$ and $\tilde{H}=P_{A}^{-1}-\varsigma I$28:    **end if**29:**end for**

Step 1 involves a broadcast by node *m* of a *single* measurement $z_{m,k}$ to its neighbours, together with the node ID *m*, to allow recipients to determine where the measurement was taken.

Step 2 involves using the collected and received measurements $\left\{z_{m,k}\right\}\cup \left\{z_{n,k}:n\in N_{m}\right\}$ to form a local pre-estimate ${\hat{\beta }}_{k}^{\left(m-\right)}$ and Hessian $H_{k}^{\left(m-\right)}$. Similar to Section 3, this is performed using an approximate Newton update on the received measurements, shown in lines 10–13 of Algorithm 2.

Step 3 involves broadcasts of $\left({\hat{\beta }}_{k}^{\left(m-\right)},H_{k}^{\left(m-\right)}\right)$ to neighbours. ${\hat{\beta }}_{k}^{\left(m-\right)}$ is a vector of *p* numbers (recall *p* is the number of radial basis functions), while $H_{k}^{\left(m-\right)}$ is a p×p matrix, where the number of basis functions *p* is usually chosen to be less than the number of nodes *M*.

Step 4 combines the pre-estimates $\left\{{\hat{\beta }}_{k}^{\left(m-\right)}\right\}\cup \left\{{\hat{\beta }}_{k}^{\left(n-\right)}:n\in N_{m}\right\}$ and Hessians to form the estimate ${\hat{\beta }}_{k}^{\left(m\right)}$, as shown in lines 18 or 21 of Algorithm 2. It does this by using one of the fusion methods in Section 4, i.e., covariance intersection or inverse covariance intersection. In the case of covariance intersection, the batch form given in (10) is used, which is less computationally intensive than combining two estimates at a time using (9) in a sequential manner, while having similar estimation performance. In the case of inverse covariance intersection, the estimates are combined two at a time using (11). (Various ways to extend inverse covariance intersection to a batch form have been proposed in [56,57]; however, we have been unable to get these methods to work in our situation). Note that (10) and (11) require error covariances, while the approximate online Newton method computes Hessians. In this paper, we will use the inverse Hessians as a surrogate for the covariances, which is motivated by the result that the Hessian matrix of the negative log-likelihood with respect to the parameters is approximately equal to the inverse of the covariance matrix [58], with equality holding for Gaussian random vectors [59]. For simplicity, we have set $\omega_{n}=1/\left(\right|N_{m}\left|+1\right),\forall n\in \left\{m\right\}\cup N_{m}$ for batch covariance intersection and ω=1/2 when combining two estimates sequentially in inverse covariance intersection. As mentioned in Section 4, one could also try to optimize $\omega_{n}$ or ω; however, this will increase the computational load of the algorithm. Furthermore, we note that the estimation diffusion scheme is actually agnostic to the particular estimate fusion method, and other consistent fusion rules can also be used. Optimizing $\omega_{n}/\omega$ and the choice of fusion method will be a topic for future investigation.

## 6. Discussions and Extensions

In this section, we will first compare the communication requirements of the two proposed schemes, and a possible way to reduce communications for measurement diffusion. We then briefly discuss how the approximate Newton update can be run in batch form, and how the algorithms can be extended to handle sensor heterogeneity.

### 6.1. Communication Requirements

In measurement diffusion, at each time step *k*, each node *m* broadcasts $C_{m,k}^{\mathrm{latest}}$ given in (4), which in general is a set containing 3M numbers, which could be large when the number of nodes *M* in the network is large. One possible way to reduce communications is as follows. If $z_{n,k_{n}}=z_{n,k_{n}-1}$, i.e., the quantized measurement at a particular node has not changed at the next time step, then the sending of $\left(n,k_{n},z_{n,k_{n}}\right)$ when broadcasting $C_{m,k}^{\mathrm{latest}}$ can be eliminated, as the neighbours of node *m* can reconstruct this information. Note, however, that this requires that measurements are collected at every time step and there are no transmission losses, so that errors do not propagate.

For estimate diffusion, Step 1 involves broadcasting of two numbers. In Step 3, the pre-estimate is a vector of *p* numbers, while the Hessian is a symmetric p×p matrix with $\frac{p(p+1)}{2}$ unique entries in general. Thus, $\frac{p(p+1)}{2}+p+2$ numbers are broadcast by each node at each time step for estimate diffusion. Note that the number of basis functions *p* will affect the quality of the field estimates, with larger values generally allowing us to capture finer field structure, but also requiring more measurements for accurate estimates [46]. For relatively simple fields, such as those considered in [15,16], one can use values as low as p=16; however, for more complicated fields one may need to use values such as p=64 or even higher (see Section 7), which will significantly increase the communication requirements.

### 6.2. Batch Newton Update

In Algorithm 1, at each *k*, an approximate Newton step is run for each new measurement in $C_{m,k}^{\mathrm{new}}$. In particular, in line 14, the operation $H^{-1}G$ needs to be carried out $|C_{m,k}^{\mathrm{new}}|$ times. A computationally less intensive alternative is to replace lines 9–16 with Algorithm 3, which computes a single approximation of the gradient and Hessian, and then performs a single approximate Newton step, on each batch of measurements in a partition of $C_{m,k}^{\mathrm{new}}$. This reduces the number of times the operation $H^{-1}G$ needs to be carried out to around $\left\lceil |C_{m,k}^{\mathrm{new}}|/N_{\mathrm{batch}}\right\rceil$, where $N_{\mathrm{batch}}$ is the batch size. In simulations, we have found this batch update to also perform well, provided $N_{\mathrm{batch}}$ is not too large, e.g., less than 100. Estimate diffusion (Algorithm 2) can also be modified to use a batch Newton update in a similar fashion.
**Algorithm 3** Batch Newton update1:Partition $C_{m,k}^{\mathrm{new}}={⋃}_{i}C_{i}^{\mathrm{batch}}$ into batches $C_{i}^{\mathrm{batch}}$ of size $\leq N_{\mathrm{batch}}$, and set $\tilde{\beta }={\hat{\beta }}_{k-1}^{\left(m\right)}$2:**for** each batch $C_{i}^{\mathrm{batch}}$ **do**3:    Set G=04:    **for** $\left(n,k_{n},z_{n,k_{n}}\right)\in C_{i}^{\mathrm{batch}}$ **do**5:        $G\leftarrow \;G+\nabla g_{n}\left(\tilde{\beta }\right)$ where $\nabla g_{n}\left(.\right)$ is computed using (7)6:        $\tilde{H}\leftarrow \;\delta \tilde{H}+\nabla^{2}g_{n}\left(\tilde{\beta }\right)$ where $\nabla^{2}g_{n}\left(.\right)$ is computed using (8)7:    **end for**8:    $H=\tilde{H}+\varsigma I$9:    $\tilde{\beta }\leftarrow \tilde{\beta }-H^{-1}G$10:**end for**11:Update ${\hat{\beta }}_{k}^{\left(m\right)}=\tilde{\beta }$

### 6.3. Heterogeneous Sensors

In this paper, we have assumed that the sensors are homogeneous, with identical capabilities. In some situations, it is more appropriate to assume heterogeneous sensors, e.g., there could be many cheap sensors with limited capabilities, plus a small number of more expensive but more capable sensors.

Suppose we consider a type of heterogeneity with regard to sensors having different quantization thresholds (number of thresholds and their values). The measurement diffusion scheme can be extended to this situation, provided each sensor knows the quantization thresholds of every other sensor in the network. While the estimate diffusion scheme can also be extended to this type of heterogeneity if each sensor knows the quantization thresholds of its direct neighbours only.

## 7. Numerical Studies

In this section, we will first describe the measures for performance evaluation of our algorithms in Section 7.1, and a centralized scheme for baseline comparison in Section 7.2, before presenting numerical examples in Section 7.3–Section 7.5. The simulations in this section are written in Python and run on an Intel Core i7 9700 PC with 16 GB of memory.

### 7.1. Performance Criteria

In this paper, we will consider two performance measures, the mean square error (MSE), and the structural similarity (SSIM) index [60]. The MSE is defined as

$$\mathrm{MSE}≜\frac{1}{|R_{d}|}\sum_{x\in R_{d}}{\left(\phi \left(x\right)-\sum_{i=1}^{p}{\hat{\beta }}_{i}K_{i}\left(x\right)\right)}^{2},$$

where ϕ(x) is the true field value at position x, and $R_{d}$ is a discretized set of points in the region R.

The structural similarity index is a measure of the similarity between two images, first introduced in [60]. Suppose the discretized set of points $R_{d}$ are located on a rectangular grid. We can then regard $\Phi =\{\phi \left(x\right):x\in R_{d}\}$ and $\hat{\Phi }=\left\{\hat{\phi }\left(x\right):x\in R_{d}\right\}=\left\{\sum_{i=1}^{p}{\hat{\beta }}_{i}K_{i}\left(x\right):x\in R_{d}\right\}$ as the image representations of the true and estimated fields, respectively, and compute the SSIM between these two images. The SSIM gives a scalar value between 0 and 1, with SSIM=1 if the two images to be compared are identical. The specific equations to compute the SSIM can be found in [60]; see also [61].

### 7.2. Centralized Scheme

For comparison with our proposed algorithms, we also consider a centralized scheme, where at each time instant *k*, all of the measurements $z_{m,k},m=1,\cdots ,M$ are transmitted to a central node or fusion centre which then performs the estimation. For large networks, this could involve information from some nodes needing to be transmitted over large distances, requiring either high transmission power or multi-hop communications. Nevertheless, the centralized scheme will serve as a useful benchmark on achievable performance. Estimation of β using the centralized scheme will be performed using the approximate Newton update, as in lines 11–14 of Algorithm 1, but now with all of the measurements $z_{m,k},m=1,\cdots ,M$ collected at time *k* being used to update ${\hat{\beta }}_{k}$.

### 7.3. Example 1: Effect of Number of Sensors on Estimated Field

In the process of forming a sensor network, knowing the minimum number of sensors needed to estimate fields to an acceptable level of accuracy could aid in reducing costs and network complexity. This subsection studies the effect that varying the number of sensors used can have on the estimated fields. We concentrate on the centralized scheme described in Section 7.2 above, in order to focus on the best performance achievable for a given number of sensors.

**Figure 2:**
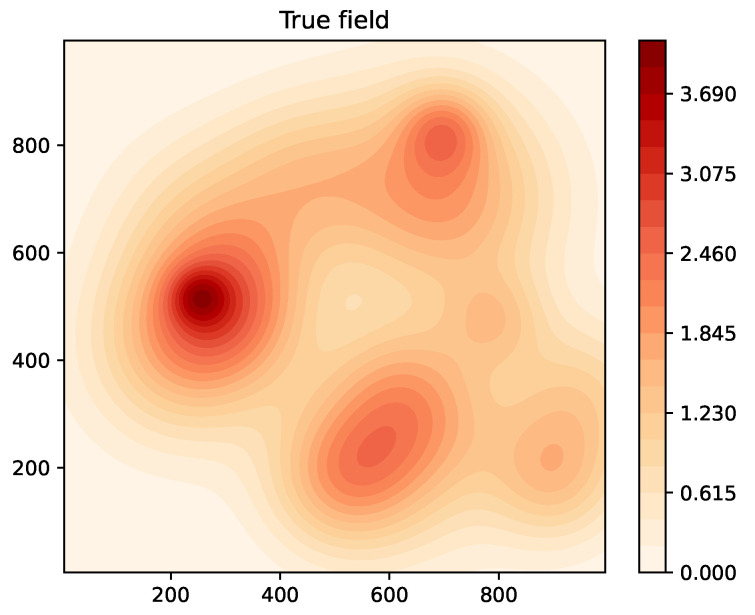
Example 1: true field.

We consider a randomly generated example true field shown in Figure 2, in the region of interest R=[0,1000]×[0,1000]. Sensor measurements are noisy, with the measurement noise assumed to be Gaussian with zero mean and variance 0.1. The quantizer is a four-level quantizer, with quantizer thresholds τ=[1,2,3]. For approximating the estimated fields, p=64 basis functions were used with $c_{i}$ spaced uniformly in a square grid within R, with $\sigma_{i}=1000/8=125$ for i=1,⋯,p. The logistic function parameter was chosen as η=5. The Levenberg–Marquardt parameter was set to ς=10, while the forgetting factor was set to δ=1. The initial estimate ${\hat{\beta }}_{0}$ was initialized by randomly choosing each component from a uniform distribution between 0 and 1.

**Figure 3:**
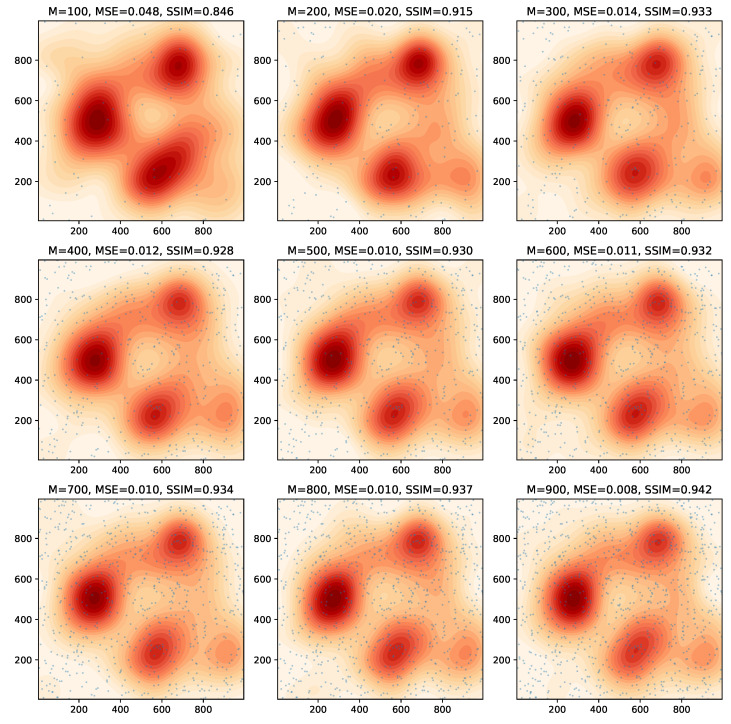
Example 1: estimated fields with varying numbers of sensors for the centralized scheme. The light blue dots represent the sensor locations.

Figure 3 depicts the estimated fields for the centralized scheme after 10 time steps, where the sensor number *M* ranges from 100 to 900 in increments of 100. The sensor locations were randomly generated, and shown as light blue dots in Figure 3. We see that a sensor number of 200–300 is already able to produce estimated fields to a reasonable level of accuracy, with the estimate reliability generally increasing as the number of sensors is increased.

### 7.4. Example 2: Estimation of Time-Varying Fields

For fields which are constantly evolving in real-time, it is crucial that the sensor system is able to adapt to these changing conditions without significant time delay. This subsection explores how each of the field estimation algorithms proposed in this paper responds to time-varying fields.

**Figure 4:**
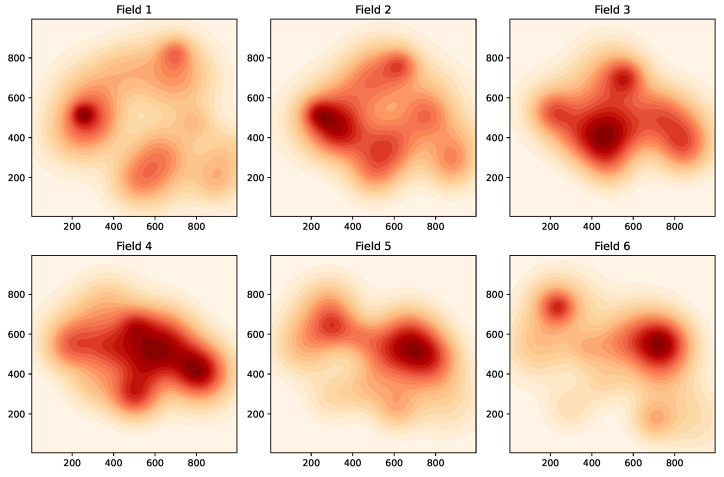
Example 2: time-varying fields.

**Figure 5:**
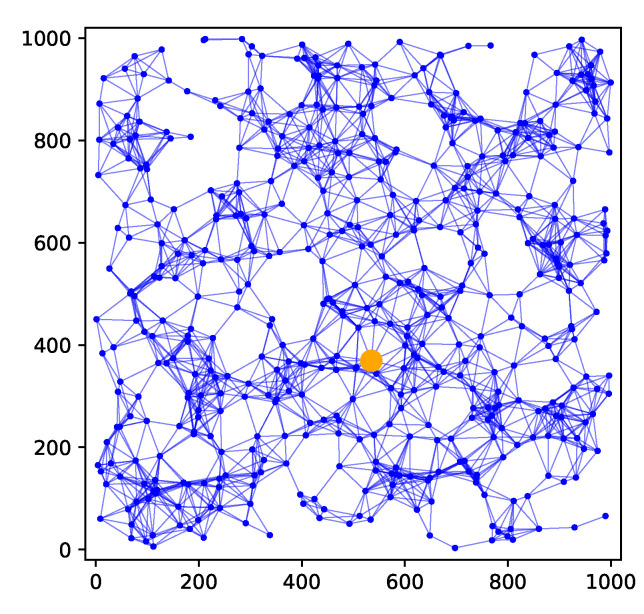
Example 2: sensor network.

For modelling the time-varying fields, we consider the six fields shown in Figure 4, where each field will be held constant for 50 time steps before switching to the next field. We will consider the sensor network shown in Figure 5, consisting of 500 randomly placed nodes. The communication range is such that two sensors within a distance of 1000/12=83.33 can communicate directly with each other. For this communication range and the chosen set of nodes, it was found that the network was connected. (In general, a larger communication range will make a network more likely to be connected; however, it will also require more transmission power). In this subsection, we will present field estimation results at the node represented by the large orange dot in Figure 5. The maximum number of hops L(m,n) (recall the notation in Section 3) between the chosen node *m* and any other node *n* in this network can be found to be 13.

In the field estimation algorithms, the Levenberg–Marquardt parameter is set to ς=10 for the centralized and measurement diffusion schemes, and ς=1 for the estimate diffusion scheme. For time-varying fields the forgetting factor is now set to δ=0.998 for centralized and measurement diffusion, and δ=0.99 for estimate diffusion. Other parameters were the same as those in Section 7.3.

**Figure 6:**
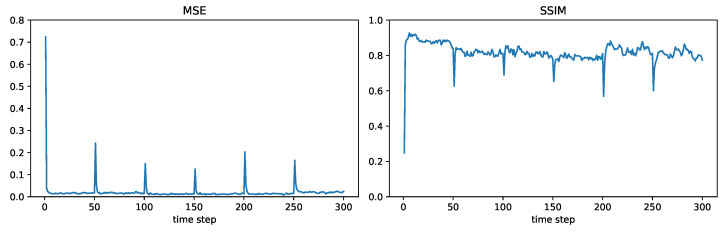
Example 2: centralized.

**Figure 7:**
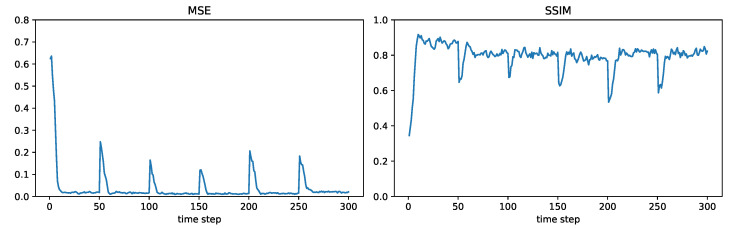
Example 2: measurement diffusion.

**Figure 8:**
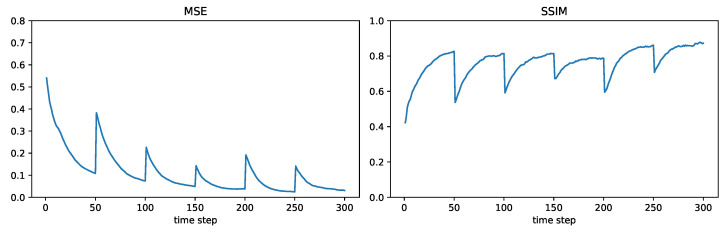
Example 2: estimate diffusion using covariance intersection.

**Figure 9:**
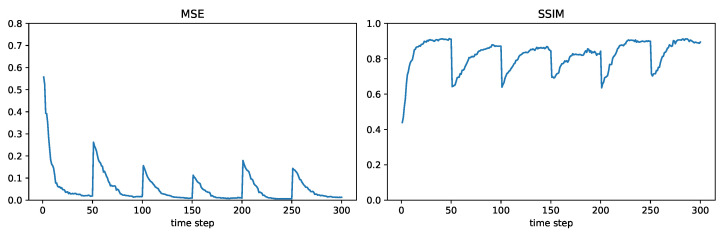
Example 2: estimate diffusion using inverse covariance intersection.

Figure 6, Figure 7, Figure 8 and Figure 9 plot the MSE and SSIM over time for centralized, measurement diffusion, estimate diffusion using covariance intersection, and estimate diffusion using inverse covariance intersection. As stated before, the fields change every 50 time steps, although we stress that the algorithms themselves do not know when the field changes occur. For the centralized scheme, the field estimate quality drops (MSE increases while SSIM decreases) immediately after the field changes, but after just 1–2 time steps (note that 500 measurements, equal to the number of sensors in the network, are collected at every time step) is able to quickly adapt to the new field. For the measurement diffusion scheme, adaptation to the field changes takes a bit longer, with the number of time steps needed to converge to a steady state roughly equal to 13, the maximum number of hops from the chosen node to any other node in the network. Comparing Figure 7 with Figure 6, we see that the MSE and SSIM values of measurement diffusion at steady state are close to that of centralized estimation. Estimate diffusion is also able to adapt to field changes, although at a slower rate than measurement diffusion, with inverse covariance intersection performing better than covariance intersection and also able to achieve steady state performance close to that of centralized estimation.

The delay in adapting to field changes is due to our assumption that each sensor communicates with its neighbours once (or twice for estimate diffusion) per time step, in order to limit the amount of communication. Whether this delay is tolerable for real time application will depend on the particular situation. Delay can be reduced if we allow for multi-hop communication within each time step, but this will increase the communication requirements. Thus, there exists a trade-off between delay and the amount of communication.

### 7.5. Example 3: Studies on a Real Dataset

**Figure 10:**
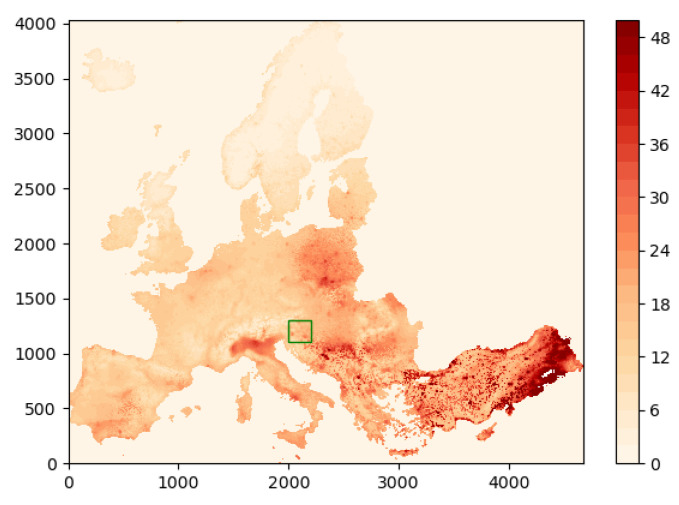
Example 3: average concentration data for PM10 air pollutants in Europe during the year 2021.

We now consider the testing of our algorithms on a publicly available dataset provided by the European Environment Agency [62]. This dataset provides interpolated average concentration data in $\mu g/m^{3}$ for PM10 (particulate matter 10 μm or less in diameter) air pollutants in Europe during the year 2021. Figure 10 shows a plot of the available data.

**Figure 11:**
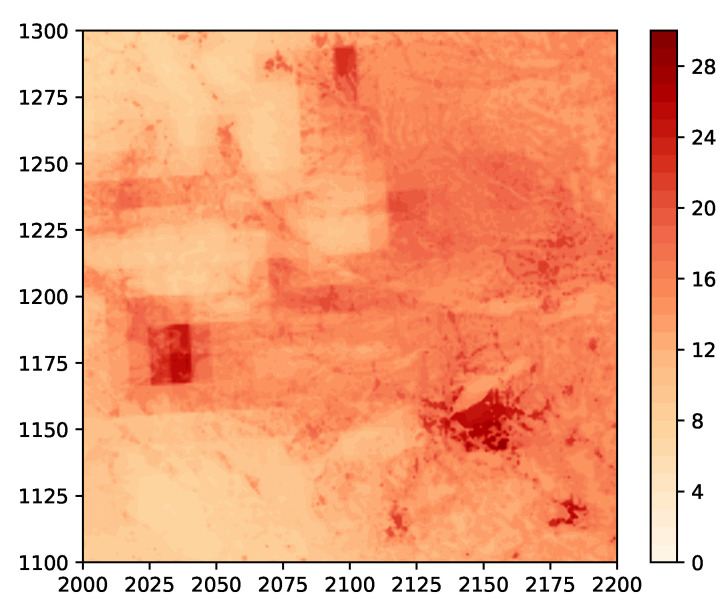
Example 3: close-up of the area bounded by the green box in Figure 10.

For testing of our field estimation algorithms, we consider the 200 km × 200 km area bounded by the green box in Figure 10. A close-up of this area is shown in Figure 11. We regard this as the true field that is to be estimated.

**Figure 12:**
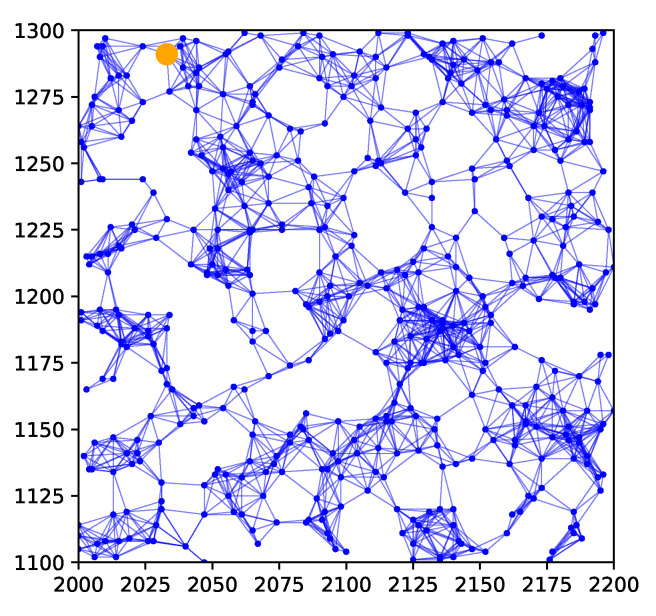
Example 3: sensor network.

We use a sensor network consisting of 500 randomly placed sensors, a plot of which is shown in Figure 12. For this network, the maximum number of hops from the orange node to any other node is 19. We assume that the sensor measurements are noisy, with the noise being Gaussian of zero mean and variance 1, with the quantizer thresholds being τ=[5,10,15,20,25]. The number of basis functions used is p=15×15=225, with $c_{i}$ uniformly spaced in a square grid within the area and with $\sigma_{i}=200/15=13.33$.

**Figure 13:**
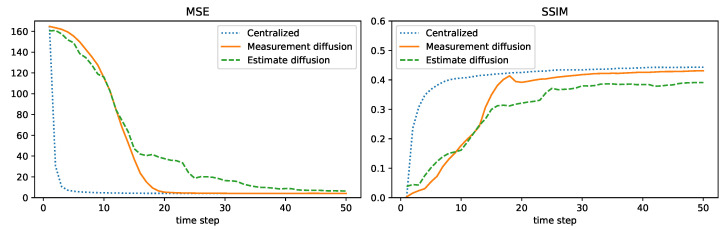
Example 3: performance comparison.

**Figure 14:**
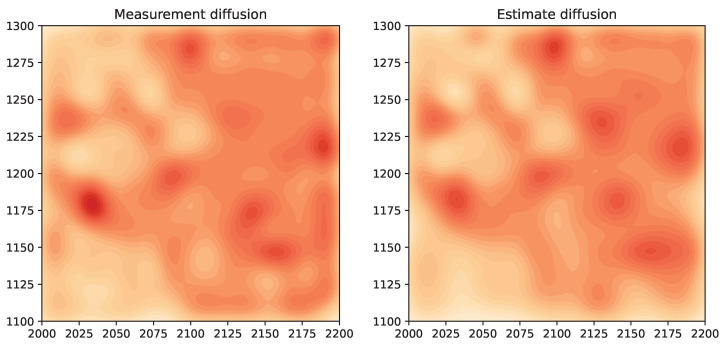
Example 3: estimated fields after 50 time steps.

We will consider results for measurement diffusion and estimate diffusion using inverse covariance intersection, with logistic function parameter η=5, and forgetting factor set to δ=1. For the orange node shown in Figure 12, Figure 13 plots the MSE and SSIM over time for the two schemes. For comparison, results for the centralized scheme are also plotted. Similar to Section 7.4, the performance of measurement diffusion reaches a steady state after around 19 time steps, which is the maximum number of hops from the orange node to any other node. While for estimate diffusion, the number of time steps needed to reach steady state is longer. For both schemes, we again see that the MSE and SSIM values at steady state are close to that of centralized estimation. The estimated fields at this node after 50 time steps are shown in Figure 14. We can see that qualitatively the estimated fields for both schemes look similar to smoothed versions of Figure 11.

## 8. Conclusions

This paper has studied the distributed estimation of scalar fields using a sensor network. Motivated by the possibility of utilizing large numbers of low cost sensors, we have considered the situation where the sensors have coarsely quantized measurements. We have proposed novel methods for distributed field estimation, which involve sharing either measurements or estimates between neighbouring sensors, and can adapt promptly to time-varying fields. Numerical studies have shown that our schemes can achieve steady state performance close to that of centralized estimation. Areas of future work include studying the impact of imperfect knowledge of sensor locations, and investigation of the use of both fixed and mobile sensors in field estimation.

## Figures and Tables

**Table 1:** Commonly used symbols.

Symbol	Description
R	Region of interest
$x_{m}$	Location of node *m*
ϕ(x)	Field value at location x
$v_{m,k}\left(x_{m}\right)$	Measurement noise of node *m* at time *k*
q(x)	Quantized value of *x*
$z_{m,k}\left(x_{m}\right)$	Quantized measurement of node *m* at time *k*
$K_{i}\left(x\right)$	*i*-th radial basis function
$N_{m}$	Neighbours of node *m*
L(m,n)	Number of hops between nodes *m* and *n*
$C_{m,k}^{\mathrm{latest}}$	Latest measurements available to node *m* at time *k* before broadcast
$C_{m,k}^{\mathrm{new}}$	New measurements for updating field estimate of node *m* at time *k*
$g_{n}\left(\beta \right)$	Per stage cost
${\hat{\beta }}_{k}^{\left(m\right)}$	Field parameter estimate of node *m* at time *k*
${\hat{\beta }}_{k}^{\left(m-\right)}$	Field parameter pre-estimate of node *m* at time *k*
$H_{k}^{\left(m-\right)}$	Approximate Hessian of node *m* at time *k*

## Data Availability

The data are available upon reasonable request.
